# Lipemia Retinalis in an Infant With a PCSK9 Variant of Familial Hypercholesterolemia: A Case Report

**DOI:** 10.7759/cureus.112310

**Published:** 2026-07-08

**Authors:** Aini Zahidah Ismail, Chi Lun Wong, Evelyn Tai Li Min

**Affiliations:** 1 Ophthalmology and Visual Sciences Department, Universiti Sains Malaysia School of Medical Sciences, Kelantan, MYS; 2 Ophthalmology Department, Hospital Kulim, Kulim, MYS

**Keywords:** familial hypercholesterolemia, infant case report, lipemia retinalis, metabolic disorder, pediatric endocrinology

## Abstract

Lipemia retinalis is a rare ophthalmic condition characterized by a milky-white appearance of the retinal vessels caused by markedly elevated blood triglyceride levels. This condition serves as an important clinical indicator of severe hyperlipidemia, particularly hypertriglyceridemia, and may signify an underlying metabolic disorder. Its ocular manifestations can serve as an early warning sign for clinicians, prompting further evaluation of the patient's lipid profile and potential underlying systemic conditions. We present a rare case of a 36-day-old male infant who was hospitalized for acute diarrhea following the initiation of formula feeding and was noted to have a milky-pink discoloration of the blood during venipuncture. An ophthalmology consultation was obtained, and fundus examination revealed bilateral dilated whitish retinal vessels and slightly palish optic discs. The anterior segments were otherwise normal, with no evidence of cataracts. Laboratory investigations demonstrated markedly elevated cholesterol (920.2 mg/dL) and triglyceride (2,742.2 mg/dL) levels, while systemic examination findings were unremarkable. No abnormalities were identified on echocardiography or abdominal ultrasonography. Genetic testing revealed a heterozygous mutation in the *PCSK9* gene, confirming the diagnosis of familial hypercholesterolemia (FH). The infant was transitioned to a specialized formula diet. At one-month follow-up, the ocular signs of lipemia retinalis had completely resolved. Ocular manifestations such as lipemia retinalis may represent an early sign of dyslipidemia and should prompt a comprehensive systemic evaluation to facilitate timely diagnosis and prevent potentially serious complications.

## Introduction

Lipemia retinalis is an ocular manifestation of severe hypertriglyceridemia, characterized by a creamy-white appearance of the retinal vessels on fundus examination. Hyperlipidemia may result from either primary or secondary causes. In neonates and young infants, inherited disorders of lipid metabolism are the most common etiology. In contrast, secondary causes are more frequently encountered in adults and include obesity, excessive alcohol consumption, poorly controlled diabetes mellitus, and the use of certain medications, such as corticosteroids and diuretics, all of which can contribute to elevated lipid levels.

Severe hyperlipidemia in neonates and infants may lead to both ocular and systemic complications. Lipemia retinalis is a characteristic ophthalmic manifestation of marked hypertriglyceridemia and may serve as an early clinical clue to an underlying metabolic disorder. Other reported complications include acute pancreatitis, hepatosplenomegaly, eruptive xanthomas, growth impairment, and, over the long term, recurrent pancreatitis and premature cardiovascular disease. Early diagnosis and appropriate treatment are therefore essential to reduce the risk of morbidity and mortality.

## Case presentation

**Figure 1:**
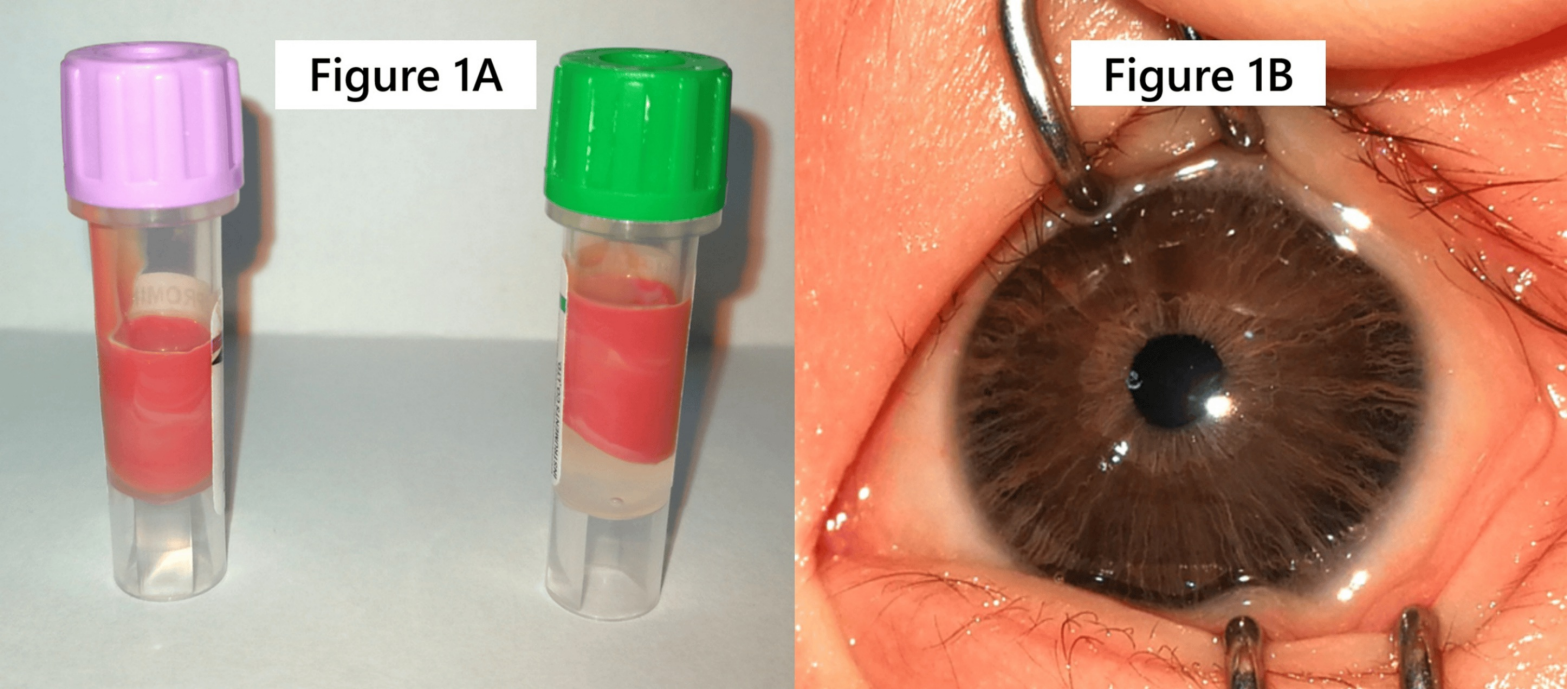
(A) Blood discoloration of the patient at presentation. (B) Anterior segment photo

A 36-day-old full-term male infant was brought to the hospital with a three-day history of diarrhea following the introduction of formula feeding. During venipuncture, a milky-pink discoloration of the blood was noted (Figure 1A), prompting further investigation. Following the laboratory findings, the patient was referred to the ophthalmology team for evaluation.

Laboratory investigations demonstrated markedly elevated total cholesterol of 920.2 mg/dL (high: ≥200 mg/dL), triglycerides of 2,742.2 mg/dL (high: ≥100 mg/dL), and high-density lipoprotein (HDL) cholesterol of 225.8 mg/dL (high: ≥60 mg/dL). Low-density lipoprotein (LDL) cholesterol could not be calculated because of the markedly elevated triglyceride level.

A complete blood count could not be performed because of the markedly lipemic nature of the blood sample. Liver function tests showed a normal total protein level of 65.4 g/L (reference range: 46-87 g/L), a slightly elevated albumin level of 67.0 g/L (reference range: 38-51 g/L), and an elevated total bilirubin level of 171.3 µmol/L (reference range: 1-20 µmol/L). The renal profile demonstrated severe hyponatremia, with a sodium level of 106.2 mmol/L (reference range: 135-145 mmol/L), and hypochloremia, with a chloride level of 80.9 mmol/L (reference range: 98-102 mmol/L). Following appropriate management, both electrolyte abnormalities resolved, with sodium and chloride levels improving to 138.4 mmol/L and 104.1 mmol/L, respectively. Potassium (3.85 mmol/L) and creatinine (177 µmol/L) levels were within the normal range. Thyroid function tests were also normal, with a free T4 level of 16.82 pmol/L and a TSH level of 4.89 mIU/L.

The infant was born to a 39-year-old mother with a 16-year history of subfertility. The mother had a history of hypothyroidism and essential hypertension and was receiving oral levothyroxine and antihypertensive medications. Her lipid profile and thyroid function tests were within the normal range. The father reported no known medical conditions, including dyslipidemia or premature cardiovascular disease. The parents were non-consanguineous and reported no family history of premature death.

**Figure 2:**
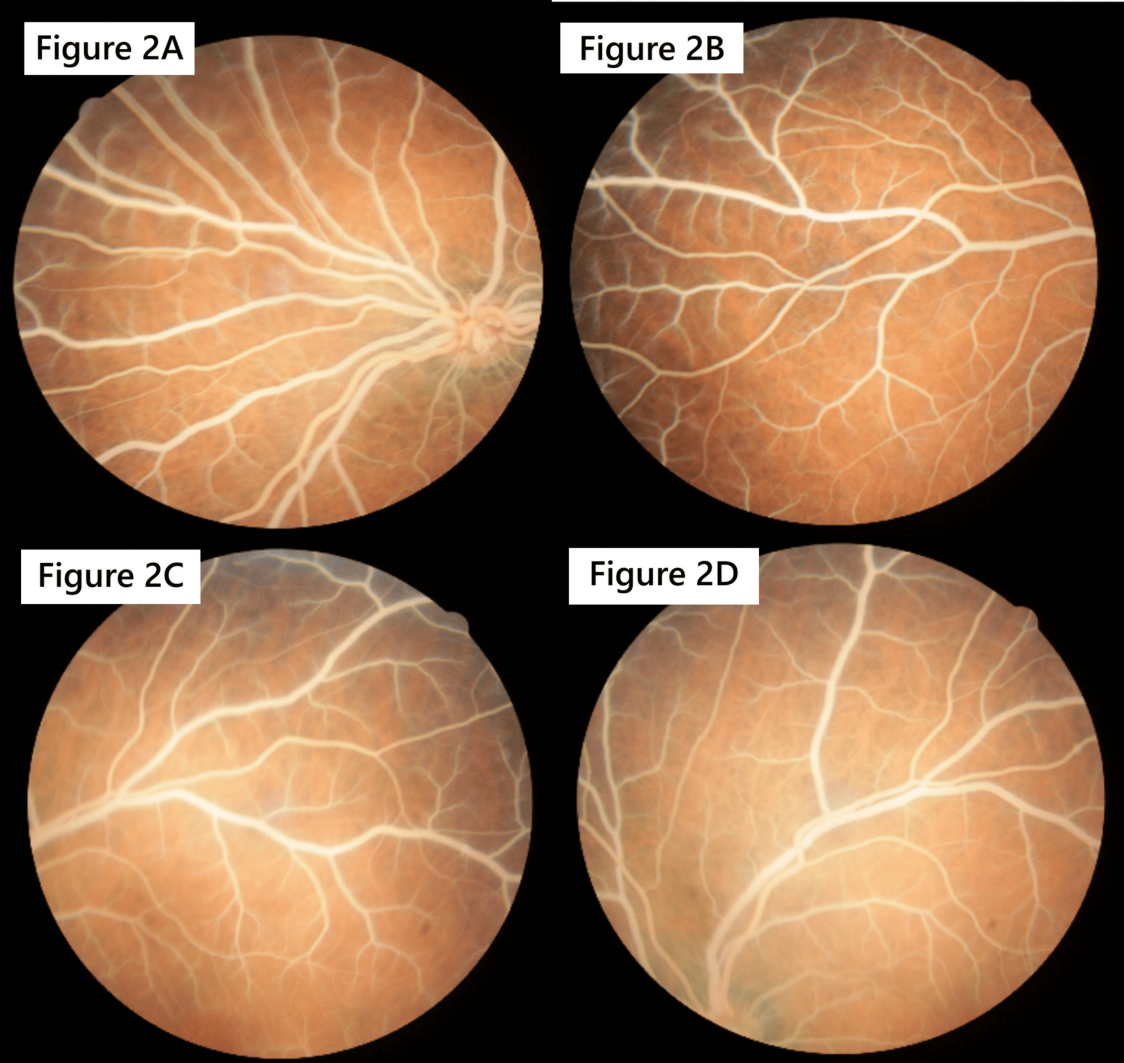
Bilateral eye fundi showing slightly dilated whitish retinal blood vessels and pale discs (A, B) Right eye. (C, D) Left eye.

During the initial ophthalmic examination, the anterior segments of both eyes appeared normal, with mild iris hypopigmentation (Figure 1B). No cataracts were detected. Fundus examination revealed prominent creamy-white retinal blood vessels that appeared mildly dilated. The retinas were flat, with no evidence of ischemic changes. There were no signs of retinal arteriosclerosis or Hollenhorst plaques. Both optic discs were of normal size but appeared slightly pale. The choroidal reflex was absent, while no abnormalities of the retinal pigment epithelium (RPE) were observed. The vitreous was clear (Figure 2).

Systemically, the infant was active and not tachypneic, although mild pallor was noted. His blood pressure was 109/63 mmHg, pulse rate was 126 beats/minute, and oxygen saturation (SpO₂) was 100% on room air. Lung auscultation was clear, and no cardiac murmurs were detected. The abdomen was soft and non-distended, with the liver palpable 1 cm below the right costal margin and no splenomegaly. Echocardiography and abdominal ultrasonography were performed, and both findings were normal.

The infant was subsequently referred to a pediatric metabolic clinic at a tertiary hospital. He was initially started on a specialized dietary regimen consisting of Portagen, a medium-chain triglyceride (MCT)-rich formula, supplemented with Carborie carbohydrate powder, which he tolerated well. Breastfeeding could not be continued because of insufficient maternal milk supply. Genetic testing revealed a heterozygous mutation in the PCSK9 gene, which is inherited in an autosomal dominant pattern, thereby confirming the diagnosis of familial hypercholesterolemia (FH).

**Figure 3:**
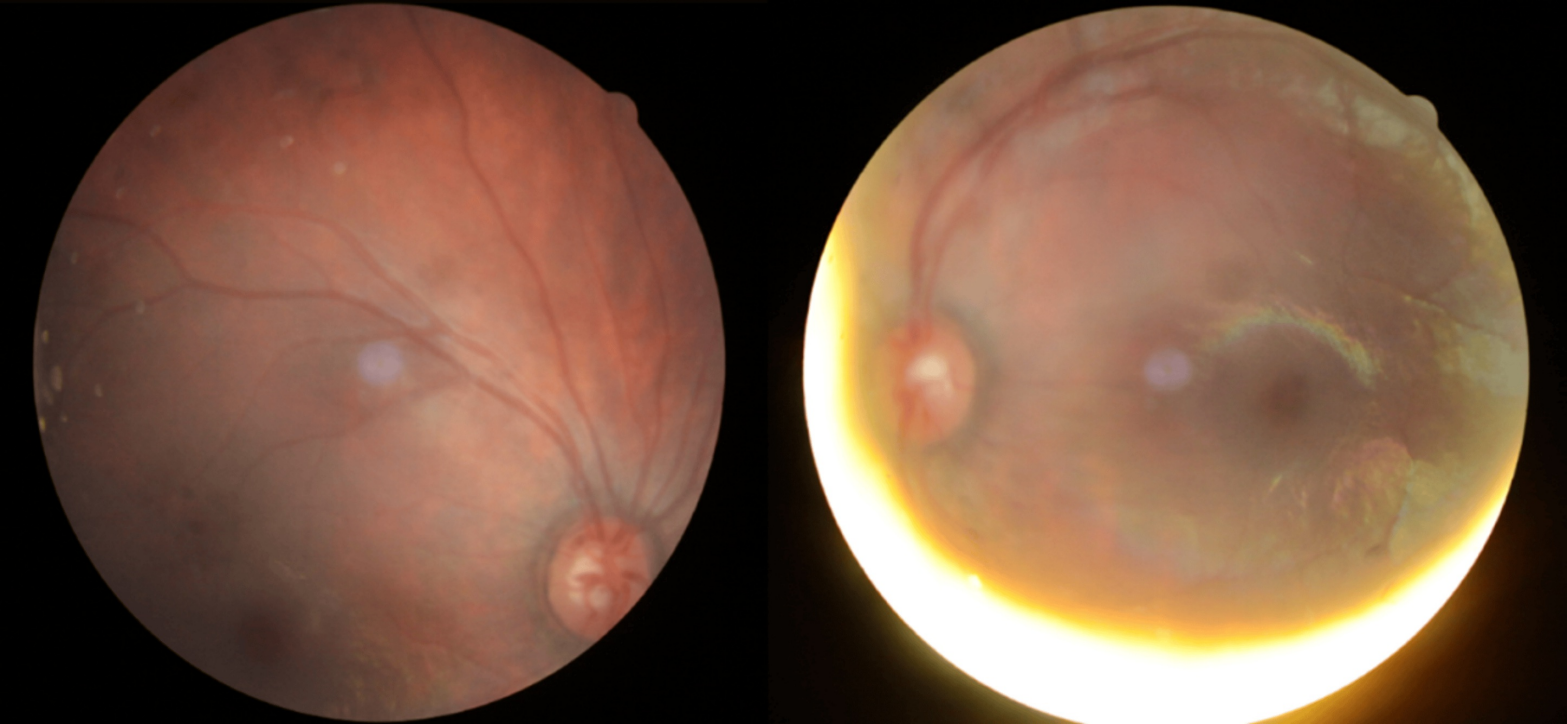
Bilateral eye fundus showing normal appearance of the retina, macula, blood vessels, and pink optic disc (one-month post-dietary adjustment)

At the follow-up visit on day 73 of life, after one month of dietary modification, fundus examination revealed complete resolution of the lipemia retinalis. Both fundi appeared normal, with healthy retinas, maculae, retinal vessels, and optic discs (Figure 3).

Over the three months following initiation of the dietary regimen, his biochemical parameters improved markedly. Total cholesterol decreased from 920.9 to 172.9 mg/dL, triglycerides decreased from 2,742.2 to 499.6 mg/dL, and HDL cholesterol decreased from 225.4 to 23.2 mg/dL. Liver and renal function tests remained within the normal ranges.

At one year of age, his best-assessed binocular visual acuity was 6/15 using the Cardiff Acuity Test at a distance of 50 cm. Cyclorefraction revealed +1.50/-0.50 × 180 in the right eye and +1.50/-0.50 × 140 in the left eye, consistent with mild bilateral hyperopia and astigmatism, which are within the normal range for his age. Fundus examination remained normal. Total cholesterol was 244.8 mg/dL, and triglyceride level was 442.8 mg/dL. Liver and renal function tests remained within the normal ranges.

**Table 1:** Lipid profile of the infant at different ages Data are presented in mg/dL. Reference values for children and adolescents: total cholesterol, acceptable <170 mg/dL, high ≥200 mg/dL; triglycerides (0-9 years), acceptable <75 mg/dL, high ≥100 mg/dL; HDL cholesterol, acceptable >45 mg/dL, low <40 mg/dL [1]. HDL: high-density lipoprotein, LDL: low-density lipoprotein.

Age	Total cholesterol (mg/dL)	Triglycerides (mg/dL)	HDL (mg/dL)	LDL (mg/dL)
38 days	920.9	2742.2	225.4	Not calculable
3 months	172.9	499.6	23.2	Not calculable
12 months	244.8	422.8	-	Not calculable

## Discussion

FH is the most common inherited disorder of cholesterol metabolism, affecting approximately 30 million individuals worldwide. It is characterized by lifelong elevation of LDL-cholesterol from birth, leading to accelerated atherosclerosis and an increased risk of premature ischemic heart disease. In severe, untreated cases, cardiovascular events may occur during childhood or early adulthood [2].

FH is most commonly caused by pathogenic variants in LDLR, APOB, or PCSK9, all of which play central roles in LDL receptor-mediated cholesterol clearance. In some individuals, elevated lipoprotein(a) (Lp(a)) may produce a phenotype that mimics FH and contributes to a substantial proportion of clinically diagnosed cases [3]. Sustained elevation of LDL-C in FH promotes early atherogenesis, underscoring the importance of timely diagnosis and intervention [3]. The diagnosis is established using clinical criteria supported by genetic testing, with cascade screening recommended for affected families. Standard treatment includes high-intensity statins with or without ezetimibe, whereas PCSK9 inhibitors are reserved for patients at very high cardiovascular risk or for those who do not achieve lipid targets despite maximal therapy. In pediatric populations, screening is recommended from the age of 5 years, with pharmacologic therapy generally initiated between 8 and 10 years of age in conjunction with lifestyle modification [3].

In the present case, the infant demonstrated markedly elevated triglyceride and cholesterol levels, and genetic testing identified a PCSK9 variant potentially associated with FH. However, FH is primarily characterized by isolated elevation of LDL-C and does not typically cause severe hypertriglyceridemia. The presence of profound hypertriglyceridemia, together with lipemia retinalis, suggests an additional or alternative disorder of triglyceride metabolism rather than classical FH alone [4]. Therefore, the PCSK9 variant may explain the hypercholesterolemic component but is unlikely to fully account for the severity of hypertriglyceridemia observed.

Severe hypertriglyceridemia in infancy most commonly reflects disorders affecting chylomicron metabolism, particularly familial chylomicronemia syndrome (FCS) [4]. FCS is a rare autosomal recessive disorder caused by biallelic loss-of-function variants in genes encoding lipoprotein lipase (LPL) or related pathway proteins, including APOC2, APOA5, GPIHBP1, and LMF1. Impaired clearance of triglyceride-rich lipoproteins results in marked chylomicron accumulation, often producing serum triglyceride levels exceeding 1,500 mg/dL and, in severe cases, reaching more than 15,000 mg/dL [4]. Clinically, FCS is characterized by eruptive xanthomas, abdominal pain, lipemia retinalis, and recurrent episodes of acute pancreatitis [5]. Given the severity of hypertriglyceridemia in this infant, FCS represents the most important differential diagnosis, and additional genetic testing targeting triglyceride metabolism pathways would be required for definitive classification.

Chylomicronemia syndrome refers to the clinical manifestation of chylomicron accumulation in plasma, typically presenting with severe hypertriglyceridemia accompanied by characteristic features such as lipemia retinalis, eruptive xanthomas, abdominal symptoms, and pancreatitis [6]. These manifestations reflect impaired clearance of triglyceride-rich lipoproteins and are most commonly observed in monogenic FCS or multifactorial chylomicronemia.

Lipemia retinalis is a rare ophthalmic manifestation of severe hyperlipidemia [7] and is strongly correlated with serum triglyceride levels [8]. It was first described by Heyl in 1880 [9]. The condition typically becomes clinically apparent when serum triglyceride levels exceed approximately 1,500 mg/dL. At extremely high triglyceride levels, often around 5,000 mg/dL or greater, the retinal vessels assume a creamy-white appearance against a salmon-pink fundus background, and differentiation between arteries and veins may become difficult because only differences in vessel caliber remain apparent [10]. Although lipemia retinalis is usually reversible with appropriate lipid control, it serves as an important clinical indicator of severe systemic dyslipidemia and an increased risk of acute pancreatitis.

Lipemia retinalis is characterized by creamy-white retinal vessels and is generally asymptomatic, although visual impairment may occur in severe cases. Other ocular manifestations associated with hyperlipoproteinemia include xanthelasma, corneal arcus (lipid keratopathy), retinal artery and vein occlusions, ischemic optic neuropathy, cataracts, and dry eye disease [8].

In summary, although the identified PCSK9 variant may contribute to the hypercholesterolemic profile, it does not adequately explain the degree of hypertriglyceridemia and the presence of lipemia retinalis observed in this infant. The clinical phenotype strongly suggests an additional underlying disorder of triglyceride metabolism, with familial chylomicronemia syndrome representing the most likely diagnosis. Comprehensive genetic testing targeting triglyceride metabolism pathways is essential to clarify the underlying etiology and guide appropriate management.

## Conclusions

This case highlights the complexity of interpreting severe dyslipidemia in infancy, particularly when genetic and biochemical findings appear discordant. Although a PCSK9 variant was identified, the clinical phenotype of extreme hypertriglyceridemia with lipemia retinalis is not characteristic of familial hypercholesterolemia alone, suggesting an additional underlying disorder of triglyceride metabolism. The findings underscore the importance of not attributing mixed or severe lipid abnormalities to a single genetic diagnosis without comprehensive phenotypic correlation. Early recognition of possible coexisting lipid disorders, particularly familial chylomicronemia syndrome, is essential, given the risk of life-threatening complications such as acute pancreatitis. This case further emphasizes the need for extended genetic evaluation and integrated lipid profiling to achieve accurate diagnosis and guide targeted management in complex pediatric dyslipidemia.
